# A novel class of lipophilic quinazoline-based folic acid analogues: cytotoxic agents with a folate-independent locus

**DOI:** 10.1038/sj.bjc.6690270

**Published:** 1999-04

**Authors:** L A Skelton, M G Ormerod, J Titley, R Kimbell, L A Brunton, A L Jackman

**Affiliations:** The CRC Centre for Cancer Therapeutics, The Institute of Cancer Research, 15 Cotswold Road, Sutton, Surrey, SM2 5NG, UK

**Keywords:** ZM198583, CB30865, TS, cell cycle, cross-resistance, folate-independent

## Abstract

Three lipophilic quinazoline-based aminomethyl pyridine compounds, which differ only in the position of the nitrogen in their pyridine ring, are described. CB300179 (2-pyridine), CB300189 (4-pyridine) and CB30865 (3-pyridine) all inhibited isolated mammalian TS with IC_50_ values of 508, 250 and 156 nM respectively. CB30865 was the most potent growth inhibitory agent (IC_50_ values in the range 1– 100 nM for several mouse and human cell types). CB300179 and CB300189 were active in the micromolar range. Against W1L2 cells, CB300179 and CB300189 demonstrated reduced potency in the presence of exogenous thymidine (dThd), and against a W1L2:C1 TS overproducing cell line. In contrast, CB30865 retained activity in these systems. Furthermore, combinations of precursors and end products of folate metabolism, e.g. dThd/hypoxanthine (HX) or leucovorin (LV), did not prevent activity. CB30865 did not interfere with the incorporation of tritiated dThd, uridine or leucine after 4 h. A cell line was raised with acquired resistance to CB30865 (W1L2:R865; > 200-fold), which was not cross-resistant to CB300179 or CB300189. In addition, W1L2:R865 cells were as sensitive as parental cells to agents from all the major chemotherapeutic drug classes. CB300179 and CB300189 induced an S phase accumulation (preventable by co-administration of dThd). No cell cycle redistribution was observed following exposure (4–48 h) to an equitoxic concentration of CB30865. In the NCI anticancer drug-discovery screen, CB30865 displayed a pattern of activity which was not consistent with known anti-tumour agents. These data suggest that CB30865 represents a class of potent potential anti-tumour agents with a novel mechanism of action. © 1999 Cancer Research Campaign


British Journal of Cancer (1999) **79**(11/12), 1692–1701
© 1999 Cancer Research Campaign
Article no. bjoc.1998.0270

### A novel class of lipophilic quinazoline-based folic acid analogues: cytotoxic agents with a folate-independent locus

**LA Skelton, MG Ormerod, J Titley, R Kimbell, LA Brunton and AL Jackman**

The CRC Centre for Cancer Therapeutics at The Institute of Cancer Research, 15 Cotswold Road, Belmont, Sutton, Surrey SM2 5NG, UK

**Summary** Three lipophilic quinazoline-based aminomethyl pyridine compounds, which differ only in the position of the nitrogen in their pyridine ring, are described. CB300179 (2-pyridine), CB300189 (4-pyridine) and CB30865 (3-pyridine) all inhibited isolated mammalian TS with $IC_{50}$ values of 508, 250 and 156 nM respectively. CB30865 was the most potent growth inhibitory agent ($IC_{50}$ values in the range 1–100 nM for several mouse and human cell types). CB300179 and CB300189 were active in the micromolar range. Against W1L2 cells, CB300179 and CB300189 demonstrated reduced potency in the presence of exogenous thymidine (dThd), and against a W1L2:C1 TS overproducing cell line. In contrast, CB30865 retained activity in these systems. Furthermore, combinations of precursors and end products of folate metabolism, e.g. dThd/hypoxanthine (HX) or leucovorin (LV), did not prevent activity. CB30865 did not interfere with the incorporation of tritiated dThd, uridine or leucine after 4 h. A cell line was raised with acquired resistance to CB30865 (W1L2:R865; > 200-fold), which was not cross-resistant to CB300179 or CB300189. In addition, W1L2:R865 cells were as sensitive as parental cells to agents from all the major chemotherapeutic drug classes. CB300179 and CB300189 induced an S phase accumulation (preventable by co-administration of dThd). No cell cycle redistribution was observed following exposure (4–48 h) to an equitoxic concentration of CB30865. In the NCI anticancer drug-discovery screen, CB30865 displayed a pattern of activity which was not consistent with known anti-tumour agents. These data suggest that CB30865 represents a class of potent potential anti-tumour agents with a novel mechanism of action.

**Keywords:** ZM198583; CB30865; TS; cell cycle; cross-resistance; folate-independent

Thymidylate synthase (TS; EC 2.1.1.45) is a critical enzyme in the de novo synthesis of thymidylate (dTTP) and has long been recognized as a target for chemotherapeutic intervention. Of interest has been a class of quinazoline-based compounds that fall into three general biochemical sub-type of antifolate TS inhibitor; those which:

1. are transported into the cell via the reduced folate carrier (RFC) and are subsequently polyglutamated by folylpolyglutamate synthetase (FPGS; EC 6.3.2.17), e.g. Tomudex™ (raltitrexed; ZD1694; reviewed in Jackman et al, 1996*a*)
2. use the RFC but which do not undergo intracellular polyglutamation, e.g ZD9331 (Jackman et al 1995*c*, 1997*a*)
3. do not interact with the RFC or FPGS, which may either retain the 'classical' (glutamate-containing) structure of antifolates (Bavetsias et al, 1997; Jackman et al, 1997*b*; Melin et al, 1997) or be 'non-classical' lipophilic derivatives (Skelton et al, 1994*a*, 1994*b*).

This latter sub-type was originally developed with the aim of achieving good TS inhibition whilst circumventing resistance due to defective RFC and/or polyglutamation mechanisms in tumours. Based on the structure of ZM198583 (2-desamino-2-methyl-$N^{10}$-propargyl-5,8-dideazafolic acid; Jackman et al, 1991), these compounds have an aminomethyl 2-, 3- or 4-pyridine in place of the glutamate residue (Skelton et al, 1997). Their highly lipophilic nature [LogP (partition coefficient) ~ 3.5] suggests that they are likely to enter the cell by passive diffusion, and the absence of the glutamate ligand precludes intracellular polyglutamation.

The pyridine derivatives were generally good inhibitors of isolated mammalian TS ($IC_{50}$ ~ 0.1–2 μM). However, although compounds with the 2- or 4-pyridine structure targeted TS (at least partially) in W1L2 human lymphoblastoid cells, those with the 3-pyridine configuration did not, since they were active in the presence of exogenous salvageable thymidine (dThd; Jackman et al, 1996*b*; Skelton et al, 1997). They were also active against cell lines resistant to antifolates due to elevated TS or dihydrofolate reductase (DHFR) enzyme levels (Skelton et al, 1997). Furthermore, there were analogues in the 3-pyridine series which were extremely potent inhibitors of in vitro tumour cell growth ($IC_{50}$ ~ 1 nM). For example, substitution with Cl or Br at the C7 position of the quinazoline ring was found to improve growth inhibitory potency against L1210 and W1L2 cell lines by ~ 100-fold (Skelton et al, 1997). A detailed account of structure–activity relationships will be given elsewhere (Skelton et al, manuscript in preparation). Thus, based on its good growth inhibitory potency (W1L2 72 h $IC_{50}$ = 0.0028 μM) and apparently folate-independent mechanism, the 7-bromo-3-pyridine derivative CB30865 (ZM242421, p-[*N*-(7-bromo-3,4-dihydro-2-methyl-4-oxoquinazolin-6-ylmethyl)-*N*-(prop-2-ynyl)amino]-*N*-(3-pyridylmethyl)benzamide; Figure 1) was selected for further in vitro studies.

*Received 9 May 1998*
*Revised 1 September 1998*
*Accepted 2 September 1998*

*Correspondence to:* LA Skelton

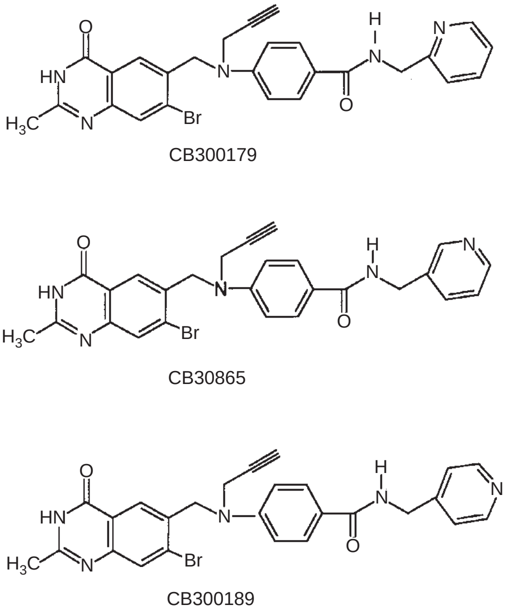

**Figure 1** Structure of CB300179 (2-pyridine), CB30865 (3-pyridine) and CB300189 (4-pyridine)

This paper presents the in vitro activity of CB30865 in terms of inhibition of both isolated and whole cell TS, growth inhibitory/cytotoxic potency (including the influence of co-incubation with combinations of end products and precursors of folate metabolism), effects on DNA/RNA/protein synthesis (as determined by incorporation of radiolabelled precursors), generation of acquired resistance and cross-resistance studies, cell cycle effects as determined by flow cytometry, and activity in the NCI in vitro anticancer drug discovery screen. Where appropriate, CB30865 is compared with its 2- and 4-pyridine equivalents CB300179 and CB300189 respectively (Figure 1). The evidence presented suggests that CB30865, differing only in the position of the nitrogen in the pyridine ring, is a potent cytotoxic agent with a unique profile of anti-tumour activity via a folate-independent target, and delayed non-phase specific effects on the cell cycle.

### MATERIALS AND METHODS

### Compounds

CB30865 (ZM242421), CB300179 (ZM249148) and CB300189 (ZM249081) were synthesized at ZENECA Pharmaceuticals, Macclesfield, Cheshire, UK. They were solubilized in dimethyl sulphoxide (DMSO) at 20 mM and dilutions were made in the same solvent. Stock solutions were stored at –20°C for a maximum of 6 months. The TS inhibitor ZD1694 (ZENECA Pharmaceuticals) and the DHFR inhibitor methotrexate (MTX; Sigma Chemical Co., Poole, Dorset, UK) were prepared as 10 mM stock solutions in 0.15 M sodium bicarbonate and diluted in RPMI-1640 (Life Technologies, Paisley, UK).

### Inhibition of mammalian L1210 TS

The inhibition of mouse TS using enzyme partially purified from the L1210:C15 TS-overproducing cell line (Jackman et al, 1986) was determined using a previously described method (Calvert et al, 1980; Jackman et al, 1984). The $IC_{50}$ against TS (defined as the concentration of drug required to inhibit the reaction rate by 50% at a (*R,S*)-5,10-$CH_2FH_4$ cofactor concentration of 200 μM) was determined. The values of $K_i$ (constant for the dissociation of the inhibitor from the enzyme-inhibitor complex; TS.dUMP.I) and $K_{ies}$ (constant for the dissociation of the inhibitor from the enzyme-inhibitor-substrate complex; TS.dUMP.5,10-$CH_2FH_4$.I) were determined by Dr W Ward at ZENECA Pharmaceuticals (Ward et al, 1992).

### Cell culture and growth inhibition assays

W1L2 human lymphoblastoid, W1L2:C1 (elevated TS; O'Connor et al, 1992) L1210 mouse leukaemia and L5178Y TK+/– mouse lymphoma (Stephens et al, 1993) cells were cultured in suspension, and CHI, A2780, PXN94, 41M, HX62, SKOV3 (human ovarian carcinoma), MCF-7 (human breast carcinoma), HT29 and SW480 (human colon carcinoma) cell lines were grown as monolayers as previously described (Jackman et al, 1990, 1995*a*). All cell lines were screened routinely for *Mycoplasma* using the Stratagene PCR method (Stratagene Ltd, Cambridge, UK).

Growth inhibition studies with suspension cells were performed as previously described (Jackman et al, 1990). Activity against the adherent cell lines was assessed by MTT assay (Twentyman et al, 1987) where cells were seeded in 96-well plates at 1000–3000 cells per well, incubated overnight and then exposed to the appropriate drug concentrations for 96–120 h. $IC_{50}$ values represented the concentration of drug required to inhibit cell growth/reduce absorbance at 540 nm to 50% control.

### Protection studies

Growth inhibition assays as described above were performed in W1L2 cells where CB30865 was incubated in the absence and presence of combinations of precursors and end products of folate metabolism. The combinations were as follows (all purchased from Sigma Chemical Co., unless otherwise stated); dThd, dThd–HX, leucovorin (LV; David Bull Laboratories, Warwick, UK), dThd–LV, *S*-adenosylmethionine (SAM), dThd–SAM, dThd–adenosine–guanosine, adenosine–guanosine–inosine, dThd–adenosine–deoxycoformycin (NCI, Bethesda, MD, USA), dThd–HX–methionine, or dThd–HX–glycine. All components were at 10 μM except HX (50 μM), LV (50 μM), SAM (15 μM), deoxycoformycin (1 μM), methionine (1 mM) and glycine (100 μM). Protection studies in the adherent cell lines were also performed by adding 10 μM dThd or 10 μM dThd–50 μM HX to the medium.

### Trypan blue viability studies

W1L2 cells ($5 \times 10^4$ cells $ml^{-1}$) were treated with 0.006, 0.03 and 0.15 μM CB30865 (equivalent to 2 ×, 10 ×, and 50 × $IC_{50}$ as determined by 72 h growth inhibition assay). Controls were treated with 0.5% DMSO. At the appropriate time, aliquots (0.5–3 ml) were counted on a Coulter counter, centrifuged at 900 ***g*** for 5 min and resuspended in phosphate-buttered saline (PBS) (pH 7.2) such that 50–200 cells could be counted on a haemocytometer. The cells were then Coulter counted again so that any losses due to centrifugation could be calculated. The PBS suspension was mixed with trypan blue (Sigma Chemical Co.) to give a final concentration of

© Cancer Research Campaign 1999

0.1%, left at room temperature for 5 min and scored on a haemocytometer. Both non-stained (viable) and stained (non-viable) cells were scored and counts were corrected for percentage recovery on centrifugation (50–100%).

### Clonogenic assay

Cytotoxic potency was determined in W1L2 cells by clonogenic assay in 96-well plates. Cells ($1–3 \times 10^5$ ml$^{-1}$) were exposed in duplicate to 0.03 μM CB30865 for 24 h in T25 Nunc tissue culture flasks. The cells were then harvested by centrifugation at 900 ***g*** for 5 min, resuspended in fresh medium and Coulter Counted. The cells were then diluted (in several steps) in 50% conditioned medium (obtained by centrifugation of cells grown to $2–5 \times 10^5$ ml$^{-1}$ at 900 ***g*** for 5 min and sterile filtered through a 0.2 μm nylon filter) to a concentration of 1.6 cells ml$^{-1}$ (controls) or 5 cells ml$^{-1}$ (treated) and 200 μl was added to each well of the plate. Control cells were seeded in seven plates (such that no fewer than 50 colonies would be counted at the end of the experiment, since the plating efficiency was 41 ± 22%) and treated cells were seeded in two plates. The plates were incubated at 37°C in a humidified atmosphere. After 2–3 weeks, colonies were visualized by the addition of 50 μl 2 mg ml$^{-1}$ MTT (Sigma Chemical Co.). All colonies consisted of > 50 cells. Colony formation of treated cells was expressed as a percentage of untreated cells.

Clonogenic assays were also performed in the HT29 human colon tumour cell line, where cells were exposed to 1 μM CB30865 for 4–72 h. With the same sample of cells, $IC_{50}$ values for CB30865 were determined in the presence and absence of 10 μM dThd by 5-day MTT assay. This was to ensure that CB30865 was not acting as a TS inhibitor at a concentration of 1 μM. Cells were seeded at $5 \times 10^4$ ml$^{-1}$ in T25 vented flasks, incubated overnight and treated in duplicate with 1 μM CB30865 for 4–72 h (controls were treated with 0.5% DMSO). At the appropriate time, the cells were harvested by trypsinization followed by centrifugation at 900 ***g*** for 10 min. The cells were then resuspended in PBS, counted on a haemocytometer and seeded in triplicate into T25 vented flasks at 300 or 3000 cells per flask. After ~ 2 weeks, colonies (> 20 cells) were visualized with 5 mg ml$^{-1}$ MTT and counted. Plating efficiencies for controls were calculated (56–99%) and colony formation in the treated samples was determined as percent of control.

### Flux through TS in intact cells

TS activity in intact W1L2 cells was determined by measuring the rate of $^3H_2O$ release, which occurs as a consequence of flux through TS in cells exposed to [5-$^3$H]dUrd (Moravek Biochemicals Inc., Brea, CA, USA). This was performed as previously described (Jackman et al, 1995*b*).

### Incorporation of radiolabelled precursors into acid-precipitable material

The rate of incorporation of [methyl-$^3$H]dThd, [5-$^3$H]Urd and L-[4,5-$^3$H]Leu (all purchased from Amersham Int. plc) into acid-precipitable material was investigated in order to determine the effects of CB30865 on DNA, RNA and protein synthesis respectively. W1L2 cells ($2 \times 10^5$ ml$^{-1}$) were exposed to 0.03 μM CB30865 for 1–14 h. After the appropriate exposure period, the cells were Coulter counted and [methyl-$^3$H]dThd, [5-$^3$H]Urd or L-[4,5-$^3$H]Leu was added to give final dThd and Urd concentrations of 0.2 μM, sp. act. 5 nCi pmol$^{-1}$, and a final Leu concentration of 2.2 nM, sp. act. 150 nCi pmol$^{-1}$. Cells were harvested ($3 \times 1$ ml aliquots) after 15, 30, 45 and 60 min incubation with radiolabel by centrifugation at 10 000 rpm for 1 min (microfuge) and resuspension in 100 μl deionized $H_2O$. DNA/RNA/protein was precipitated on ice by the addition of 1.5 ml ice-cold 2% v/v PCA. After 10 min on ice, the acid-precipitable material was washed in 1.5 ml 2% vv PCA, resuspended in 1 ml 10% v/v PCA, and heated to 80°C for 60 min. The samples were then counted in Ultima Gold Scintillant, and the data fitted to a linear regression. The slope was proportional to the rate of incorporation of the radiolabelled precursor in terms of dpm per number of cells. This was standardized to give a rate in pmol min$^{-1}$ per $10^6$ cells.

### Generation of acquired resistance to CB30865 and cross-resistance studies

A cell line was raised with acquired resistance to CB30865, denoted W1L2:R865, by stepwise selection over ~ 9 months. Initially, W1L2 cells were incubated with 0.002 μM CB30865 (72 h $IC_{50}$ = 0.0028 μM), which was raised in small increments (< twofold) until the cells became resistant to 0.03 μM. When larger increments were attempted, the cells did not survive. This is in contrast to generation of a resistant line to the 2-pyridine derivative, CB300179 (Kobayashi et al, 1995), where the concentration of the latter could be raised in increments of at least twofold. The generated W1L2:R865 cell line was routinely cultured in 0.03 μM CB30865 under the same conditions as W1L2 cells, with cells being grown in the absence of CB30865 for ~ 2 weeks before an experiment.

The activity of a number of clinical and experimental anticancer and cytotoxic agents (refer to Table 2) against W1L2 and W1L2:R865 cells was determined within parallel experiments by 72 h growth inhibition assay (Coulter counts). CB30865 was included in every experiment as an internal control. The resistance factor (RF) for each compound ($IC_{50}$ W1L2:R865 cells/$IC_{50}$ W1L2 cells) was then calculated. All compounds were purchased from Sigma Chemical Co. except trimetrexate (TMQ; supplied by the NCI), metoprine (supplied by Wellcome Labs, Beckenham, Kent, UK), lometrexol (supplied by Eli Lilly Laboratories, Indianapolis, IN, USA), 2′-deoxycoformycin (supplied by the NCI), doxorubicin (purchased from Farmitalia CarloErba Ltd, Milton Keynes, Bucks, UK), cisplatin (synthesized by Johnson Matthey Technology Centre, Reading, Berks, UK), bleomycin (purchased from Lundbeck Ltd, Luton, Beds, UK), trimelamol (synthesized by Warner Lambert, Ann Arbor, MI, USA, under contract to the NCI and supplied as a gift to the NCI) and taxol (supplied by Bristol-Myers-Squibb, Princeton, NJ, USA).

### Flow cytometry

DNA histograms were obtained as previously described (Ormerod, 1994). W1L2 (at $1–2 \times 10^5$ ml$^{-1}$) and CH1 (at $5 \times 10^4$ ml$^{-1}$) cells were incubated with drug over a 48-h time course. For protection studies, the cells were incubated with both drug and 10 μM dThd or 10 μM dThd–50 μM HX. Data were collected on one of two systems: (1) an Ortho Cytofluorograph 50H equipped with

© Cancer Research Campaign 1999

a Spectraphysics argon-ion laser. The Ortho Cytofluorograph was associated with an Ortho 2150 computer system, and the data were transferred to an IBM compatible PC and analysed using software written by Dr Michael G Ormerod; (2) a Coulter EPICS Elite ESP equipped with the laser described above. Data were analysed using Multicycle software by Phoenix Flow Systems (Oregon, USA).

### NCI anticancer drug discovery screen

Screening was carried out by the Developmental Therapeutics Program, Division of Cancer Treatment, Diagnosis and Centres, NCI. In this way, the activity of CB30865 was determined against panels of human tumour cell lines by 48-h sulforhodamine B (SRB) assay as previously described (Boyd, 1989; Boyd et al, 1992; Monks et al, 1991). For each cell line, three response parameters were calculated; $GI_{50}$, TGI and $LC_{50}$. The $GI_{50}$ represents the concentration of CB30865 required to reduce net cell growth to 50% control after 48 h. The TGI represents the concentration of CB30865 required to achieve a percentage growth of zero after 48 h. The $LC_{50}$ represents the concentration of CB30865 required to cause a net 50% reduction in SRB staining (i.e. cellular protein) after 48 h (i.e a net 50% loss of cells). These parameters were ranked in terms of deviation from the mean (denoted mean graphs; Paull et al, 1989). 'COMPARE' analysis was used to compare the spectrum of activity of CB30865 with that of standard anticancer agents of known mechanisms of action, generating Pearson correlation coefficients which describe the degree of similarity of CB30865 to these agents.

### RESULTS

### Inhibition of isolated TS

CB300179 (2-pyridine), CB30865 (3-pyridine) and CB300189 (4-pyridine) inhibited isolated mammalian TS with $IC_{50}$ values of 508, 156 and 250 nM respectively. The $K_i$ and $K_{ies}$ values for CB300179 were 160 and 350 nM, respectively, indicating mixed non-competitive inhibition. CB30865 also inhibited TS in a non-competitive manner ($K_i$ = 110 nM ≅ $K_{ies}$). ZM198583 is reported to inhibit TS with an $IC_{50}$ of 50 nM ($K_i$ = 10 nM; Jackman et al, 1991).

**Table 1** Growth inhibitory activity of the 3-pyridine derivative CB30865 against some rodent and human tumour cell lines[a]

| Cell line | Origin | CB30865 $IC_{50}$ (μM) |
|---|---|---|
| W1L2 | Human lymphoblastoid | 0.0028 ± 0.00050[b] |
| L1210 | Mouse leukaemia | 0.074 ± 0.033[c] |
| L5178Y | Mouse lymphoma | 0.013 ± 0.0089 |
| MCF-7[d] | Human breast tumour | 0.0025 ± 0.00065 |
| HT29[d] | Human colon carcinoma | 0.026 ± 0.0058 |
| SW480[d] | Human colon carcinoma | 0.024 ± 0.00080 |
| Various | Human ovarian carcinoma | 0.0021 to > 1[e] |

[a]Activity determined by growth inhibition assays as described in Materials and Methods. [b] ± SD ($n \geq 3$). [c]No protection with dThd at a single concentration equivalent to 10 × $IC_{50}$ (Skelton et al, manuscript in preparation. [d]Also tested in the presence of dThd or dThd/HX (refer to text). [e]Refer to Figure 2 and text.

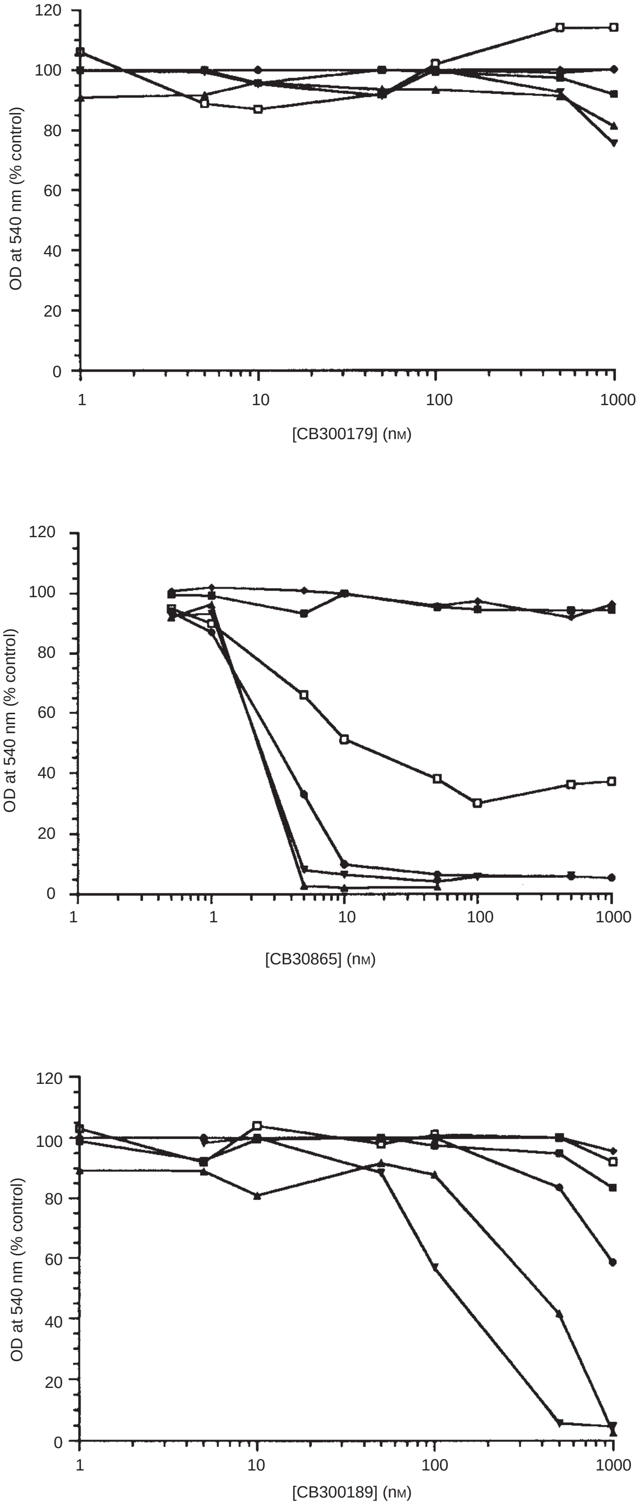

**Figure 2** Representative growth inhibition curves from human ovarian carcinoma cells exposed to CB30865, CB300179 or CB300189. Cells were incubated with the indicated concentrations of drug for 96 h and growth inhibition was assessed by MTT assay: 41M (■); A2780 (▲); CH1 (▼); HX62 (◆); PXN94 (●); SKOV3 (□). CB300189 inhibited CH1, A2780 and PXN94 cell growth with $IC_{50}$ values of 0.43 ± 0.18, 1.1 ± 0.56 and 1.5 μM (mean, $n$ = 2) respectively. CB30865 inhibited CH1, A2780, PXN94 and SKOV3 cell growth with $IC_{50}$ values of 0.0021 ± 0.00044, 0.0025 ± 0.00040, 0.0054 ± 0.0023 and 0.0099 ± 0.0029 μM respectively

© Cancer Research Campaign 1999

### Inhibition of cell growth and protection studies

The activity of CB300179, CB300189 and CB30865 against W1L2 human lymphoblastoid cells was 0.58, 0.27 and 0.0028 μM respectively (published as part of a Table in Skelton et al, 1997). CB30865 was further tested against a range of other rodent and human tumour cell lines (Table 1). Some cell lines were exquisitely sensitive (e.g MCF-7, $IC_{50}$ = 0.0025 μM), whilst others were relatively resistant (e.g L1210, $IC_{50}$ = 0.074 μM).

With regard to the panel of ovarian carcinoma cell lines (Figure 2), CB300179 was relatively inactive with all $IC_{50}$ values > 1 μM. CB300189 was active at the submicromolar level only against two cell lines (CH1 and A2780). In contrast, four of the ovarian cell lines were highly sensitive to CB30865, with $IC_{50}$ values of 2.1–9.9 nM (CH1, A2780, PXN94 and SKOV3).

### Cell viability studies and clonogenic assay

Trypan blue staining was used as an indication of cell viability following incubation of W1L2 cells with 0.006–0.15 μM CB30865 over ~ 2 weeks. At a concentration of 0.006 μM (2 × $IC_{50}$ as determined by 72 h growth inhibition assay), the viable cell concentration increased at the same rate as controls over 24 h. After this time, there was some growth up to 48 h but it was less than controls, and the number of viable cells gradually reduced to < 3% of total cells after 168 h (~ 12-fold lower than the initial viable cell count). At the higher concentrations of 0.03 and 0.15 μM (equivalent to 10 and 50 × 72 h $IC_{50}$ respectively), proliferation was again similar to controls for 24 h (although cells treated with 0.15 μM CB30865 grew slightly less), after which time there was no further increase in cell number. Population viability decreased to below the initial viable cell count after 71 h exposure and was eventually reduced to zero after 168 h. Although CB30865-treated cell populations were still largely viable after 24 and 48 h, it would appear that they may be destined to die since a 24 h exposure to 0.03 μM CB30865-inhibited colony formation by 95 ± 4.5% compared to untreated controls (data not shown).

Clonogenic assays were also performed in HT29 human colon carcinoma cells at a concentration of 1 μM CB30865. It was confirmed that CB30865 did not act via TS at this concentration by 5-day MTT assay in the absence and presence of dThd (data not shown). Colony formation was comparable to controls after 4 h exposure, but was reduced to ~ 60% after 24 h exposure. A greater

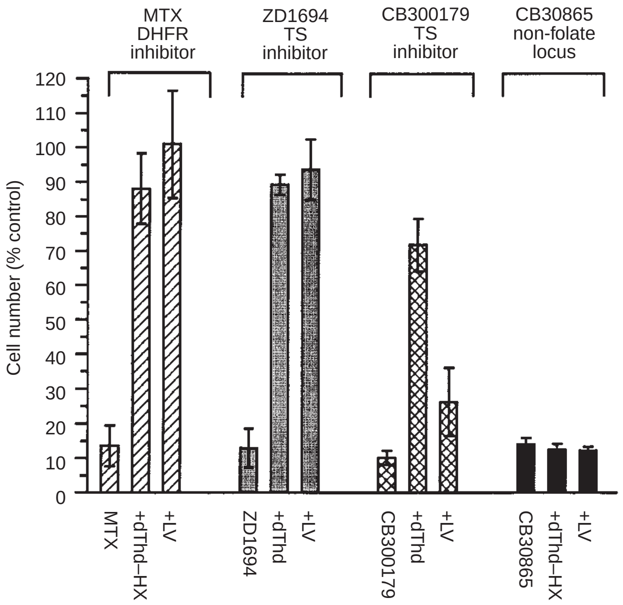

**Figure 4** W1L2 protection studies with MTX, ZD1694, CB300179 and CB30865. Cells were co-incubated for 72 h with CB30865 (0.03 μM), CB300179 (5.8 μM), MTX (0.1 μM) or ZD1694 (0.04 μM) and protection combinations shown. Inhibition of cell growth was assessed by Coulter counts

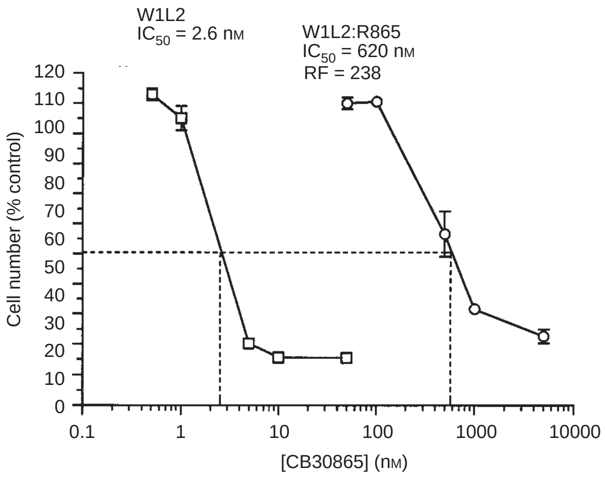

**Figure 5** Representative growth inhibition curves demonstrating differential activity of CB30865 against W1L2 and W1L2:R865 (acquired resistance) cell lines. Activity was determined by 72-h growth inhibition assay (Coulter counts). Resistance factors (RF) calculated as described previously. Error bars represent duplicate values within the same experiment

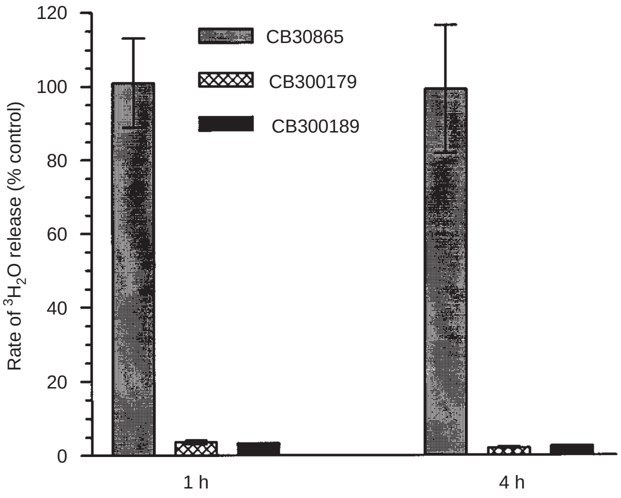

**Figure 3** Effect of CB30865, CB300179 and CB300189 on flux through TS in intact W1L2 cells. Cells were treated with 0.03 μM CB30865, 5.0 μM CB300179 or 2.7 μM CB300189 for 1 or 4 h and TS inhibition was determined by the rate of $^3H_2O$ release that occurs as a consequence of flux through TS in cells incubated with [5-$^3$H]dUrd. Mean control values were 1.45 ± 0.322 (1 h) and 1.38 ± 0.278 (4 h) pmole $min^{-1}$ per $10^6$ cells. Experiments were performed at least in duplicate. Error bars represent S.D.

*© Cancer Research Campaign 1999*

**Table 2** Cross-resistance studies in W1L2 versus W1L2:R865 cell lines[a]

| Class: Agent | $IC_{50}$ (μM) | | |
|---|---|---|---|
| | W1L2 | W1L2:R865 | RF[b] |
| Antimetabolites | | | |
| CB300179 | 0.58 ± 0.15[c] | 0.82, 1.4 | 1.9 |
| CB300189 | 0.24 ± 0.017 | 0.47 ± 0.11[d] | 2.0 |
| 5-Fluorodeoxyuridine | 0.0049 ± 0.0013 | 0.0060 | 1.2 |
| 5-Fluorouracil | 5.4 | 8.0 | 1.5 |
| 6-Mercaptopurine | 4.7 | 3.0 | 0.6 |
| Cytosine arabinoside | 0.042, 0.047 | 0.020, 0.020 | 0.5 |
| Metoprine | 0.031 ± 0.0030 | 0.019, 0.052 | 1.2 |
| Deoxycoformycin | 0.00042 | 0.00012 | 0.3 |
| Lometrexol | 0.032, 0.062 | 0.060, 0.094 | 1.8 |
| MTX | 0.023, 0.0096 | 0.023 | 1.4 |
| Mycophenolic acid | 0.23 | 0.27 | 1.2 |
| Thioguanine | 0.14 | 0.13 | 0.9 |
| Trimetrexate | 0.0030 | 0.0040 | 1.3 |
| ZD1694 (Tomudex™) | 0.0043 ± 0.00048 | 0.0046, 0.0030 | 1.1 |
| DNA interactive agents | | | |
| Doxorubicin | 0.013 ± 0.0045 | 0.016, 0.026 | 1.6 |
| Bleomycin | 1.0, 3.7 | 0.32, 0.32 | 0.5 |
| Camptothecin | 0.0020 | 0.0026 | 1.1 |
| Chlorambucil | 2.1, 1.7 | 4.2, 2.0 | 1.6 |
| Cisplatin | 0.76, 0.48 | 0.52, 0.70 | 1.0 |
| Etoposide | 0.017 | 0.019 | 1.1 |
| Mitomycin C | 0.019, 0.018 | 0.047, 0.025 | 1.9 |
| Trimelamol | 26 | 31 | 1.2 |
| Spindle poisons | | | |
| Taxol | 0.0066 | 0.0072 | 1.1 |
| Vinblastine | 0.0058 | 0.0090 | 1.6 |
| Protein kinase/phosphatase inhibitors | | | |
| Okadaic acid | 0.014, 0.014 | 0.0068, 0.0076 | 0.5 |
| Sodium vanadate | 8.0 | 7.0 | 0.9 |
| Staurosporine | 0.031 | 0.050 | 1.6 |
| Metabolic poisons | | | |
| Antimycin A | 0.0072 | 0.0063 | 0.9 |
| Oligomycin | 0.0083 | 0.0087 | 1.1 |
| Potassium cyanide | 190, 145 | 190, 210 | 1.2 |
| Rotenone | 0.020 | 0.015 | 0.8 |
| Miscellaneous/undefined | | | |
| Aphidicolin | 0.049 | 0.062 | 1.3 |
| Bromophenacyl bromide | 1.5, 0.36 | 1.3, 0.6 | 1.0 |
| $Ca^{2+}$ ionophore A23187 | 0.088 | 0.080 | 0.9 |
| Chloroquine | 12, 11 | 24, 7.2 | 1.4 |
| Cyclocreatine | 11400 | 11400 | 1.0 |
| Cycloheximide | 0.33 | 0.39 | 1.2 |
| Cytochalasin B | 0.94 | 1.4 | 1.5 |
| Dimethylamiloride | 69 | 70 | 1.0 |
| Ouabain | 0.052 | 0.032 | 0.6 |
| Sulfonylurea LY295501 | 40 | 34 | 0.9 |
| Tubercidin | 0.10, 0.68 | 0.31, 0.092 | 2.4 |

[a]Activity was determined by 72 h growth inhibition assay (Coulter counts). [b]Resistance factors (RF) calculated as described in Materials and Methods. [c]± SD (n ≥ 3).[d]not significant (*P* > 0.05, Student's *t*-test).

degree of inhibition of colony formation was observed after 48 h (~ 70%) and 72 h (> 99%). Thus against these cells, cytotoxicity was time-dependent.

### Inhibition of TS in intact W1L2 cells

At concentrations equivalent to 10 × 72 h $IC_{50}$, the 2- and 4-pyridine derivatives CB300179 (5 μM) and CB300189 (2.7 μM) respectively caused a reduction in the rate of product ($^3H_2O$) formation (i.e. TS activity) to < 4% control after 1 and 4 h exposure (Figure 3). In contrast, TS activity was maintained at control levels after 1 and 4 h exposure to 0.03 μM CB30865.

### Incorporation of radiolabelled precursors into acid precipitable material

The incorporation of [methyl-$^3$H]dThd, [5-$^3$H]Urd and L-[4,5-$^3$H]Leu was unaffected after W1L2 cells were exposed to

0.03 μM CB30865 for 1 and 4 h (data not shown). There was a ~50% reduction in [methyl-$^3$H]dThd and [5-$^3$H]Urd, but not L-[4,5-$^3$H]Leu, incorporation after 14 h exposure. This suggests that CB30865 is not acting directly through DNA, RNA or protein synthesis mechanisms.

### Protection studies

With regard to HT29 and SW480 colon carcinoma cells, the potency of CB30865 was not reduced in the presence of salvageable dThd ($IC_{50}$ = 0.029 ± 0.0091 and 0.029 ± 0.018 μM respectively). Similarly, the potency of CB30865 against MCF-7 cells was not reduced in the presence of dThd/HX ($IC_{50}$ = 0.0028 ± 0.00036 μM). Against the sensitive ovarian cell lines shown in Figure 2, potency was also maintained in the presence of dThd–HX (data not shown). An extended range of protection studies was carried out using W1L2 cells. Cells were co-incubated with CB30865 and various combinations of precursors and end products of folate metabolism in order to determine whether the growth inhibitory effects of this compound could be prevented. At a concentration of 0.03 μM CB30865, cell number was reduced to 10–15% control after 72 h exposure even in the presence of dThd–HX or LV (Figure 4). In addition, CB30865 retained this potency in the presence of various combinations of dThd, HX, LV, SAM, adenosine, guanosine, inosine, deoxycoformycin, methionine and glycine (data not shown). These results suggest that CB30865 exerts its growth inhibitory effect via a folate-independent target. In contrast, the growth inhibitory effect of an equitoxic concentration of CB300179 was reduced in the presence of dThd, indicative of TS locus (Figure 4). In contrast, the activities of ZD1694 (TS inhibitor) and MTX (DHFR inhibitor) were prevented by dThd or dThd–HX respectively.

### Generation of acquired resistance and cross-resistance studies

The W1L2:R865 cell line was > 200-fold resistant to CB30865, and resistance was stable for ~ 2 months (data not shown). Representative growth inhibition curves for parent and resistant lines are shown in Figure 5. The doubling time of the resistant line was similar to the W1L2 parent (~ 18 h). Although the W1L2:R865 cell line was cross-resistant in varying degrees to other 3-pyridyl quinazolines which were structurally similar to CB30865 (data not shown), no cross-resistance to any other agent tested, of known or undefined mechanism of action, was observed (Table 2).

### Analysis of cellular DNA using propidium iodide staining and flow cytometry

In W1L2 cells, at concentrations equivalent to ~ 10 × 72 h $IC_{50}$ CB300179 (5.8 μM) and CB300189 (2.7 μM) induced cell cycle effects consistent with TS inhibition, and thus DNA synthesis. Representative histograms are shown in Figure 6A and B. After only 4 h exposure, there was a decrease in the number of cells in G2/M compared to controls. This is due to the division of cells in G2, without the concomitant entry of S phase cells into G2. After 16 h exposure, cells began to build up in early S phase, and at this time ≥ 80% of the population was in S phase. There was a slow progression of cells into mid S phase over the next 32 h. As expected of TS inhibitors, when the cells were continuously co-incubated with 10 μM dThd, or 10 μM dThd–25 μM HX, none of the above effects were observed.

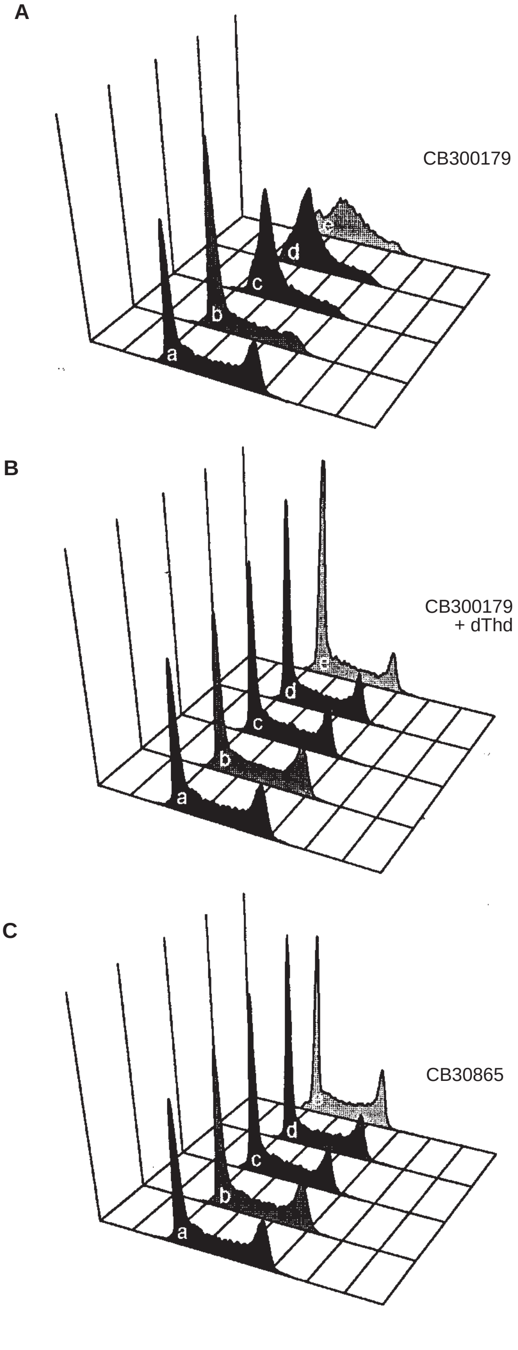

**Figure 6** DNA histogram analysis of W1L2 cells exposed to 5.8 μM CB300179 in the absence (**A**) and presence (**B**) of dThd or 0.03 μM CB30865 (**C**) over 48 h. Histograms obtained by fixing cells in 70% ethanol, incubating with RNAse and propidium iodine for 30 min, and flow cytometric analysis: a, control; b, 4 h; c, 16 h; d, 24 h; e, 48 h

A different DNA histogram pattern was obtained with CB30865, where this compound appeared to have very little effect on the cell cycle (Figure 6C). For the 48 h that the cycle was followed, the profiles were similar to controls, with no appreciable changes in distribution. Similar DNA histograms were observed

*© Cancer Research Campaign 1999*

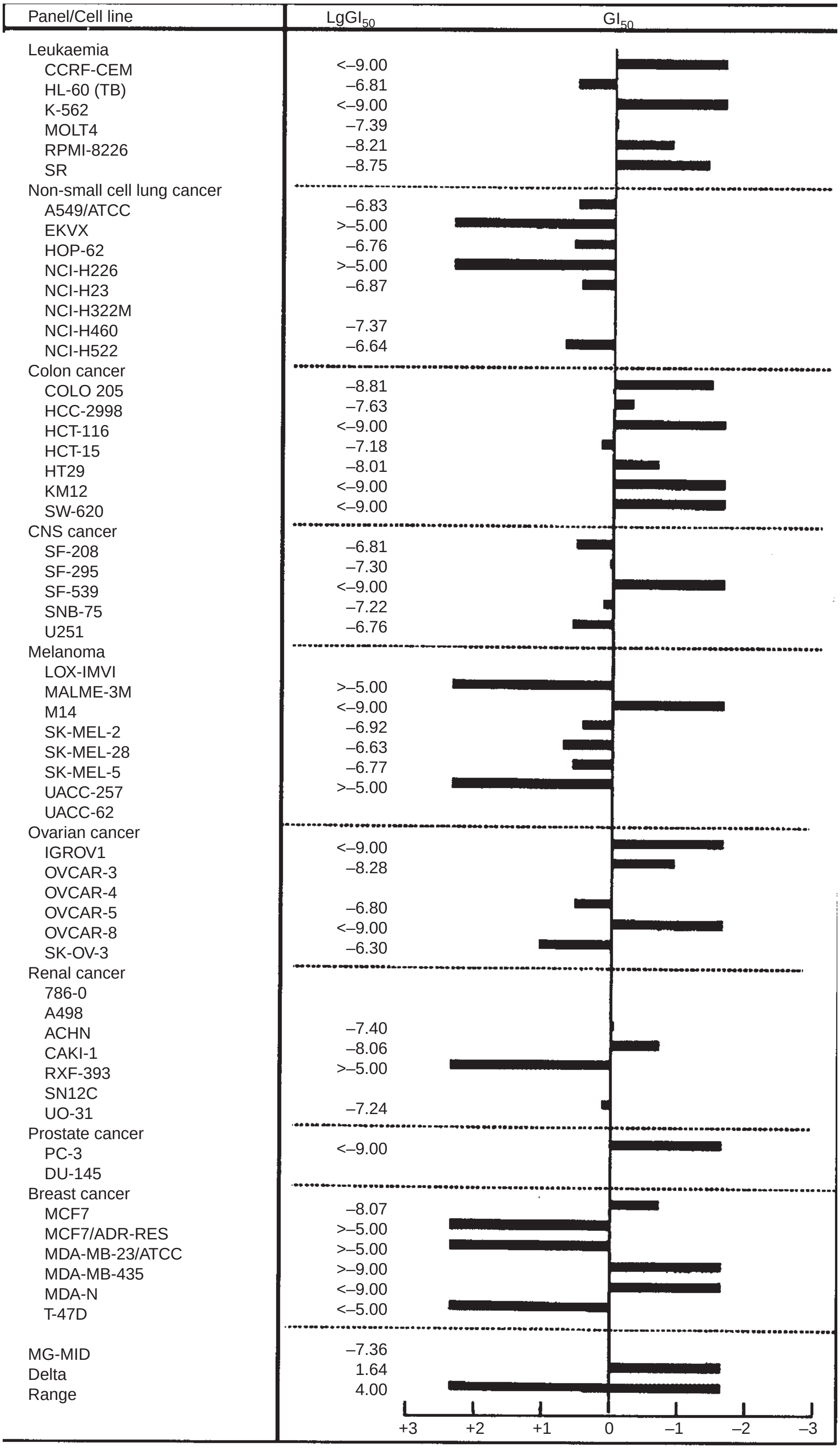

**Figure 7** Mean $GI_{50}$ graph showing the activity of CB30865 against the human tumour cell lines of the NCI in vitro drug discovery screen. Graph generated as described in ‘Materials and Methods’. Bars extending to the right represent sensitivity of the cell line to CB30865 in excess of the average sensitivity of all tested cell lines. Since the bar scale is logarithmic, a bar 2 units to the right implies CB30865 gave a $GI_{50}$ for the cell line at a concentration 1/100th the mean concentration required over all cell lines, and thus the cell line is unusually sensitive. Bars extending to the left correspondingly imply sensitivity less than the mean. CNS, central nervous system

*© Cancer Research Campaign 1999* 

with the CH1 human ovarian carcinoma cell line, where 0.003, 0.015 and 0.075 μM CB30865 (equivalent to 2×, 10× and 50 × $IC_{50}$ as determined by 4-day MTT assay) did not induce any gross changes in cell cycle distribution (data not shown).

### Activity and 'COMPARE' analysis of CB30865 in the NCI anticancer drug discovery screen

A representative mean $GI_{50}$ graph for CB30865 is shown in Figure 7. CB30865 demonstrated particularly good activity against leukaemia and colon tumour cell line panels, with $GI_{50}$ values from < 1 to 150 nM. Other panels, such as breast, ovarian and renal cancers, as well as melanomas, showed variable responses to CB30865, with $GI_{50}$ values ranging from < 0.1 to > 1000 nM. With respect to COMPARE analysis, all Pearson correlation coefficients were < 0.55 (data not shown), and specifically, CB30865 gave a correlation of 0.21 against the lipophilic TS inhibitor AG337. These data are supportive of a non-TS locus for CB30865 and suggest that this compound does not have a pattern of activity consistent with known anti-tumour agents.

However, in some cell lines, growth inhibition values observed in this screen ($GI_{50}$) do not compare with other data presented in this manuscript. For example, CB30865 inhibited SKOV3 cells with an $IC_{50}$ of 10 nM and a $GI_{50}$ of ~ 500 nM. This could relate to the short 48 h exposure used in the NCI screen. Indeed, CB30865 was also tested using a 6-day assay, and all $GI_{50}$ values were < 10 nM.

### DISCUSSION

The in vitro effects of the structurally related folate-based compounds CB300179 (2-pyridine), CB30865 (3-pyridine) and CB300189 (4-pyridine) have been compared. Whilst CB300179 and CB300189 displayed relatively poor growth inhibitory potency, CB30865 was highly active in vitro against a number of human tumour cell lines. However, despite being a potent inhibitor of isolated TS ($K_i$ = 110 nM), the data are consistent with CB30865 not inhibiting a known folate-dependent site within cells. This is shown by its activity in the presence of salvageable dThd and combinations of other precursors and end products of folate metabolism. Furthermore, only CB30865 did not inhibit the flux through TS in intact W1L2 cells. These, and other data, demonstrate that CB30865 displays activity not typical of compounds that target folate metabolism. For example, flow cytometric studies demonstrated that DNA histograms showing typical effects of TS inhibition were produced by CB300179 and CB300189 which were preventable by co-incubation with dThd. DNA histograms derived from CB30865-treated W1L2 cells (at a cytotoxic concentration) showed no gross changes in distribution compared to untreated cells. However, such analyses are limited in that they produce a static profile at a given time and provide no information about cycling kinetics. Extensive flow cytometric studies using the continuous BrdUrd labelling technique (which is a dynamic measurement), are published elsewhere (Skelton et al, 1998). These studies revealed that CB30865 had a major cell cycle effect, in that cells arrested simultaneously in all phases of the cycle after 20–24 h exposure. Therefore, CB30865 induced cell cycle effects inconsistent not only with those of antimetabolites, but also with other antineoplastic agents (which tend to cause phase-specific arrest; Charcosset, 1986). For example, antifolates such as methotrexate, trimetrexate and CB3717 ($N^{10}$-propargyl-5,8-dideazafolic acid) tend to cause accumulation of cells in S phase or at the G1/S interphase (Taylor et al, 1981; Hook et al, 1986; Lorico et al, 1988). Agents which disrupt formation of the mitotic spindle, such as the *Vinca* alkaloids, arrest cells in G2/M.

Secondly, a CB30865-resistant cell line (W1L2:R865) has been generated which appears to possess a novel mechanism of resistance. This cell line was sensitive to the large number of clinical and experimental agents tested, including drugs from all the major chemotherapeutic classes. This suggests that CB30865 may act via a unique mechanism. In contrast, a cell line generated with acquired resistance to the 2-pyridine derivative (CB300179) was cross-resistant to other TS inhibitors, due to an increased TS activity (~ 20-fold; Kobayashi et al, 1995). This cell line was not cross-resistant to CB30865.

Furthermore, against the human tumour cell lines of the NCI in vitro anticancer drug-discovery screen, CB30865 displayed a pattern of activity which was not consistent with known anti-tumour agents (i.e. it was 'COMPARE-negative'). This again suggests that CB30865 acts via a novel locus with respect to established anticancer agents.

Further characterization of the CB30865-resistant cell line should provide information as to the mechanism of action of this compound. Using comparative genomic hybridization, preliminary results indicate that this resistant line has a multiple-fold amplification on chromosome 7q22, and it is believed that identification of the gene(s) involved will lead to an understanding of the compound's action.

In summary, CB300179, CB300189 and CB30865 represent a series of related compounds differing only in the position of the nitrogen in the pyridine ring, and this appears to dictate the mode of action in cells. In particular, the 3-pyridine analogue CB30865 has been shown to be one of a class of highly potent, potential anticancer agents, whose mechanism of action in vitro is inconsistent with current anticancer agents. With cancer research currently focused on the development of more effective drugs acting via novel targets, the 3-pyridine derivative series, exemplified by CB30865, represents an exciting prospect.

### ACKNOWLEDGEMENTS

The authors wish to thank Dr Tom Boyle, Prof. Ken Harrap, and Prof. Paul Workman for helpful discussions. Supported by ZENECA Pharmaceuticals (LAS studentship) and by a project grant from the Cancer Research Campaign, UK.

### REFERENCES

Bavetsias V, Marriott JH, Melin C, Kimbell R, Boyle FT and Jackman AL (1997) Synthesis and antitumour activity of cyclopenta[*g*]quinazoline-based antifolates, a novel class of thymidylate synthase (TS) inhibitors. *Br J Cancer* **75** (Suppl 1): 24

Boyd MR (1989) Status of the NCI preclinical antitumor drug discovery screen: implications for selection of new agents for clinical trial. In *Cancer: Principles and Practice of Oncology Updates*, Vol. 3, De Vita VT, Hellman S and Rosenberg SA (eds), pp. 1–12. Lippincott: Philadelphia

Boyd MR, Paull KD and Rubinstein LR (1992) Data display and analysis strategies for the NCI disease-orientated in vitro antitumour drug screen. In *Cytotoxic Anticancer Drugs: Models and Concepts for Drug Discovery and Development*, Valeriote FA, Corbett T and Baker L (eds), pp. 11–34. Kluwer Academic Publishers: Amsterdam

Calvert AH, Jones TR, Jackman AL, Brown SJ and Harrap KR (1980) An approach to the design of antimetabolites active against cells resistant to conventional agents illustrated by quinazoline antifolates with $N^{10}$-substitutions. In *Advances in Tumor Prevention, Detection and Characterisation*, Vol. 5, Davis W, Harrap KR and Stathopoulos G (eds), pp. 272–283. Excepta Medica: Amsterdam

*© Cancer Research Campaign 1999*

Charcosset JY (1986) Effects of antineoplastic agents on the cell cycle progression. *Biol Cell* **58**: 135–138

Hook KE, Nelson JM, Roberts BJ, Griswold DP and Leopold WR (1986) Cell cycle effects of trimetrexate (CI-898). *Cancer Chemother Pharmacol* **16**: 116–120

Jackman AL, Calvert AH, Hart LI and Harrap KR (1984) Inhibition of thymidylate synthetase by the new quinazoline antifolate CB3717; enzyme purification and kinetics. In *Purine Metabolism in Man IV, Part B*: *Biochemical, Immunological and Cancer Research*, de Bruyn C, Simmonds H and Miller M (eds), pp. 375–378. Plenum Press: New York

Jackman AL, Alison DL, Calvert AH and Harrap KR (1986) Increased thymidylate synthase in L1210 cells possessing acquired resistance to $N^{10}$-propargyl-5,8-dideazafolic acid (CB 3717): development, characterisation, and cross-resistance studies. *Cancer Res* **46**: 2810–2815

Jackman AL, Taylor GA, O'Connor BM, Bishop JA, Moran RG and Calvert AH (1990) Activity of the thymidylate synthase inhibitor 2-desamino-$N^{10}$-propargyl-5,8-dideazafolic acid and related compounds in murine (L1210) and human (W1L2) systems in vitro and in L1210 in vivo. *Cancer Res* **50**: 5212–5218

Jackman AL, Newell DR, Gibson W, Jodrell DI, Taylor GA, Bishop JA, Hughes LR and Calvert AH (1991) The biochemical pharmacology of the thymidylate synthase inhibitor, 2-desamino-2-methyl-$N^{10}$-propargyl-5,8-dideazafolic acid (ICI 198583). *Biochem Pharmacol* **42**: 1885–1895

Jackman AL, Kelland LR, Kimbell R, Brown M, Gibson W, Aherne GW, Hardcastle A, Boyle-FT (1995*a*) Mechanisms of acquired resistance to the quinazoline thymidylate synthase inhibitor ZD1694 (Tomudex) in one mouse and three human cell lines. *Br J Cancer* **71**: 914–924

Jackman AL, Kimbell R, Brown M, Brunton L, Boyle FT (1995*b*) Quinazoline thymidylate synthase inhibitors: methods for assessing the contribution of polyglutamation to their in vitro activity. *Anti-Cancer Drug Design* **10**: 555–572

Jackman AL, Kimbell R, Brown M, Brunton L, Harrap KR, Wardleworth JM and Boyle FT (1995*c*) The antitumour activity of ZD9331, a non-polyglutamatable quinazoline thymidylate synthase inhibitor. In *Purine and Pyrimidine Metabolism in Man*. *Advances in Experimental Medicine and Biology*, Vol. 370, Sahota A and Taylor M (eds), pp. 185–188. Plenum Press: New York

Jackman AL, Boyle FT and Harrap KR (1996*a*) Tomudex™ (ZD1694): from concept to care, a programme in rational drug discovery. *Invest New Drugs* **14**: 305–316

Jackman AL, Skelton LA, Kimbell R, Hughes LR and Boyle FT (1996*b*) Lipophilic quinazoline analogues of folic acid with 3-pyridylamide replacing the glutamate: evidence for a non-folate locus. *Proc Amer Assoc Cancer Res* **37**: 394

Jackman AL, Kimbell R, Aherne GW, Brunton L, Jansen G, Stephens TC, Smith MN, Wardleworth JM and Boyle FT (1997*a*) Cellular pharmacology and in vivo activity of a new anticancer agent, ZD9331: a water-soluble, nonpolyglutamatable, quinazoline-based inhibitor of thymidylate synthase. *Clinical Cancer Res* **3**: 911–921

Jackman AL, Kimbell R, Melin C, Boyle FT, Marriott J and Bavetsias V (1997*b*) Folate-based (acidic) thymidylate synthase (TS) inhibitors with activity independent of the reduced-folate carrier (RFC) and folylpolyglutamate synthetase (FPGS). *Proc Amer Assoc Cancer Res* **38**: 162

Kobayashi H, Takemura Y, Miyachi H, Skelton LA and Jackman AL (1995) Effect of hammerhead ribozyme against thymidylate synthase on the cytotoxicity of thymidylate synthase inhibitors. *Jpn J Cancer Res* **86**: 1014–1018

Lorico A, Toffoli G, Boiocchi M, Erba E, Broggini M, Rappa G and D'Incalci M (1988) Accumulation of DNA strand breaks in cells exposed to methotrexate or $N^{10}$-propargyl-5,8-dideazafolic acid. *Cancer Res* **48**: 2036–2041

Melin C, Kimbell R, Bavetsias V, Marriott JH, Boyle FT and Jackman AL (1997) In vitro activity of the cyclopenta[g]quinazolines, a novel class of thymidylate synthase (TS) inhibitors. *Br J Cancer* **75** (Suppl 1): 24

Monks A, Scudiero D, Skehan P, Shoemaker R, Paull K, Vistica D, Hose C, Langley J, Cronise P, Vaigro-Wolff A, Gray-Goodrich M, Campbell H, Mayo J and Boyd M (1991) Feasibility of a high-flux anticancer drug screen using a diverse panel of cultured human tumour cell lines. *J Natl Cancer Inst* **11**: 757–776

O'Connor BM, Jackman AL, Crossley PH, Freemantle SE and Calvert AH (1992) Human lymphoblastoid cells with acquired resistance to C2-desamino-C2-methyl-$N^{10}$-propargyl-5,8-dideazafolic acid (ICI 198583); a novel folate-based TS inhibitor. *Cancer Res* **52**: 1137–1143

Ormerod MG (1994) Analysis of DNA-general methods. In *Flow Cytometry*: *A Practical Approach*, 2nd edn, Ormerod MG (ed), pp. 118–135. Oxford University Press: New York

Paull KD, Shoemaker RH, Hodes L, Monks A, Scudiero DA, Rubinstein L, Plowman J and Boyd MR (1989) Display and analysis of patterns of differential activity of drugs against human tumour cell lines: development of mean graph and COMPARE algorithm. *J Natl Cancer Inst* **81**: 1088–1092

Skelton LA, Kimbell R, Brunton L, Boyle FT and Jackman AL (1994*a*) Lipophilic inhibitors of thymidylate synthase: 2-pyridyl quinazolines. *Br J Cancer* **69** (Suppl XXI): 41

Skelton LA, Kimbell R, Brunton LA, Boyle FT and Jackman AL (1994*b*) 2-Pyridyl quinazolines as inhibitors of thymidylate synthase. *Proc Amer Assoc Cancer Res* **35**: 301

Skelton LA, Kimbell R, Boyle FT, Jackman AL (1997) Aminomethyl pyridine analogues of the quinazoline antifolate ICI 198583 with a folate-independent locus of action. In *Chemistry and Biology of Pteridines and Folates*, Pfleiderer W and Rokos H (eds), pp. 209–212. Blackwell Science: Berlin

Skelton LA, Ormerod MG, Titley J and Jackman AL (1998) Cell cycle effects of CB30865 – a lipophilic quinazoline-based analogue of the antifolate TS inhibitor, ICI 198583, with an undefined mechanism of action. *Cytometry* **33**: 56–66

Stephens TC, Smith MN, Waterman SE, McCloskey ML, Jackman AL and Boyle FT (1993) Use of murine L5178Y lymphoma thymidine kinase mutants for in vitro and in vivo antitumour efficacy evaluation of novel thymidylate synthase inhibitors. In *Chemistry and Biology of Pteridines and Folates*, Ayling J, Nair MG and Baugh CM (eds), pp. 589–582. Plenum Press: New York.

Taylor GA, Jackman AL, Balmanno K, Hughes LR and Calvert AH (1989) Estimation of the in vitro and in vivo inhibitory effect of antifolates upon thymidylate synthase (TS) in whole cells. In *Purine and Pyrimidine Metabolism in Man VI, Part B*, Mikanagi K, Nishioka K and Kelley WN (eds), pp. 383–388. Plenum: New York

Taylor IW and Tattersall MNH (1981) Methotrexate cytotoxicity in cultured human leukaemic cells studied by flow cytometry. *Cancer Res* **51**: 1549–1558

Twentyman PR and Luscombe M (1987) A study of some variables in a tetrazolium dye (MTT) based assay for cell growth and chemosensitivity. *Br J Cancer* **56**: 279–285

Ward WHJ, Kimbell R and Jackman AL (1992) Kinetic characteristics of ICI D1694: a quinazoline antifolate which inhibits thymidylate synthase. *Biochem Pharmacol* **43**: 2029–2031

*© Cancer Research Campaign 1999*
